# Action of Koch’s Lymph in Bovine Tuberculosis

**Published:** 1891-06

**Authors:** 


### ACTION OF KOCH'S LYMPH IN BOVINE TUBERCULOSIS.

The first application of Koch's lymph in veterinary medicine has been made at the Veterinary Institution at Dorpat, by Professor Gutman.

The lymph was injected into the costal region, behind the shoulder, of three cows, in which tuberculosis was diagnosed from physical examination of the chest, and presence of bacilli in the milk and in the discharges. The day before the operation the rectal temperature was taken every two hours from 8 A. M., until 6 P. M., and on the next day every hour, night and day.

Cow No. 1. Nine years old, weighs 374, kilograms has coughed for a month, bacilli in bronchial secretion and in the milk; no apparent lesions in the mammæ. She received on December 25, 1890, at 9 A. M., an injection of one decigram of lymph, and the evening of Dec. 28 a second injection of three decigrams of lymph.

Cow No. 2. Five years old, weighs 376 kilograms, has coughed three months, has the lymphatics of the throat and of the inguinal region enlarged; nothing abnormal on percussion of the chest, on auscultation exaggerated vesicular murmur, bacilli not found, she received on Dec. 25, at 9 A. M. an injection of two decigrams of lymph.

Cow No. 3. Fifteen years old, emaciated, weighs 262 kilograms, pleuretic friction on the left side, strong vesicular murmur both sides; tubercular bacilli in the bronchial secretions. This animal received on Dec. 25, at 9 A. M. an injection of three decigrams of lymph.

Koch's lymph causes in tuberculous cattle a sensible elevation of temperature. This elevation occurred in the three animals about *eleven hours* after the inoculation. The intensity and duration of the reaction was in direct ratio to the quantity of lymph injected. In the first case the temperature reached 40° c. (104° F.); in the second, 40.8° c. (105.1° F.); in the third, 41.3° c. (106.3° F.). The duration of the fever was four hours in the first, nine hours in the second, and ten hours in the third. In the first cow, which received a second injection on the nights of Dec. 28–29, although the quantity was tripled, the reaction showed in eleven hours, and was less intense than that which followed the first. In two healthy (as shown by autopsy afterwards) bulls, injection of lymph, made at the same time as that on the diseased cows, caused no sensible elevation of temperature. As shown by these experiments that Koch's lymph causes fever in tuberculous cattle and does not in healthy cattle, it may be considered an efficacious means of diagnosis in tuberculosis of the bovine race, especially in difficult cases.

The Imperial Sanitary Bureau of Germany, published the results of experiments made with Koch's lymph in bovine tuberculosis. These experiments were made by Roeckl and Schütz, under the supervision of Dr. Koch, on, first; two cows diagnosed as tuberculous by Professor Eggeling; second, a healthy heifer, as control subject. The injections consisted of a half cubic centimetre of lymph in 4 1-2 cubic centimetres of a half per cent. watery solution of phenic acid. In the first tuberculous cow the injection was made at 8 A. M. January 24, her temperature was then 38.8° c. at 9 P. M. it reached 40° c. at 3 A. M. 40.3° c. at 8 A. M.; and lowered to 39° c. at noon. The animal was then slaughtered and showed lungs and thoracic ganglia containing a mass of tubercular matter and numerous bacilli.

The second cow, with a temperature of 38.1° c. was injected at 9 A.M. At 8 P.M., the temperature reached 42.2° c.; at 1 A. M. 40.9° c. at 7 A. M., 40.1° c. and at 3 P. M., it had returned to 38.9° c. On slaughter, the cow was found tuberculous in the lungs, and lymphatic ganglia of the chest, liver and spleen. There were numerous bacilli in the caseous matter, contained in the pulmonary cavities. Several hours after the injections there was redness, tumefaction and sensibility around the point of puncture. The third animal, healthy heifer, with a temperature of 38.8° c. showed no reaction after injection. The autopsy showed her to be perfectly sound.

Dr. Sticker, Veterinarian at Cologne, injected four tuberculous cows with 1 c. c. of lymph and obtained reaction in from seven to nine hours. The autopsy on one, made three days later, showed recent irritation of the lungs. Two other cows with chronic affections, not tubercular, received the same injection and showed no reaction.

At the Veterinary School at Toulouse, Drs. Labat and Conte, obtained similar results. An injection of two decigrams in a tuberculous cow raised the temperature from 38.4° c. to 39.8° c. in nine hours; thirty-six hours later an injection of four decigrams raised from normal to 40° c. in twelve hours. The inoculated points showed local inflammatory reaction. The autopsy confirmed the diagnosis. The conclusion is the same as in the other experiments; that Koch's provokes an important febrile reaction in tuberculous animals.

Professor Degive* draws the following conclusions: "Supposing that Koch's lymph may be an efficacious curative remedy in a majority of cases, it would not be an economical method to use with our domestic animals. The cures would never be sufficiently radical, nor sure, to give the guarantee demanded by public hygeine. No one could assume that the animal apparently cured, had not latent spots and stupefied tuberculous germs, waiting only for an accidental cause to bring them to life, and a new proliferation with all the dangers of the disease. It is wiser to treat tuberculous animals as now required by the laws of Sanitary Police."

"But if Koch's lymph appears to have little value as a veterinary therapentic agent, it still seems to be of importance as a reactive, as a means of diagnosis of tuberculosis."

Further experiments should be made to determine, first; in what doses Koch's lymph should be used to obtain a general reaction, of desirable degree; second, if the general augmentation of temperature is a constant effect of the inoculation; third, if this reaction will occurr in other chronic affections, which may be confounded with tuberculosis.

A commission at the Alfort Veterinary School, has undertaken a series of experiments, as has Professor Degive, which may determine these points.

*Revue Vétérinaire* April, 1891.

---

* Annales de médecine vétérinaire, 1891, p. 85.

